# Tailored interventions to improve adherence to medication in elderly patients with Parkinson’s disease: a study protocol for a randomized controlled trial (AdhCare)

**DOI:** 10.1186/s13063-023-07663-9

**Published:** 2023-10-12

**Authors:** Sarah Mendorf, Ulrike Teschner, Thomas Lehmann, Tino Prell, Hannah Maria Mühlhammer

**Affiliations:** 1 Department of Neurology, Jena University Hospital, Jena, Germany; 2 Institute of Medical Statistics, Computer and Data Sciences, Jena University Hospital, Jena, Germany; 3 Department of Geriatrics, Halle University Hospital, Halle, Germany

**Keywords:** Adherence, Intervention, Randomization, Parkinson’s disease, Chronic diseases, Medication

## Abstract

**Background:**

Nonadherence to medication is a major issue in patients with chronic disorders such as Parkinson’s disease (PD). Many interventions for increasing adherence have been tested, and these have shown weak-to-moderate efficiency. Although the best methods to improve adherence remain unclear, it is reasonable to use tailored interventions instead of the “one-size-fits-all” approach.

**Methods:**

A randomized, controlled, triple-blinded trial in elderly patients with PD is conducted to test the efficacy of AdhCare, a tailored intervention to enhance adherence compared with that achieved with routine care (64 participants per arm). Motor function, quality of life, and adherence measures will be assessed at baseline and at 3 and 6 months of follow-up. The type of intervention depends on the main personal reason for nonadherence (e.g., forgetting to take the medication or poor knowledge about the medication).

**Discussion:**

The results of this study will provide valuable information for health professionals and policymakers on the effectiveness of tailored interventions in elderly patients with PD.

**Trial registration:**

German Clinical Trials Register DRKS00023655. Registered on 24 February 2021. Last update on 22 March 2023.

## Administrative information

Note: the numbers in curly brackets in this protocol refer to SPIRIT checklist item numbers. The order of the items has been modified to group similar items (see http://www.equator-network.org/reporting-guidelines/spirit-2013-statement-defining-standard-protocol-items-for-clinical-trials/)

**Table:** 

Title {1}	**Tailored interventions to improve adherence to medication in elderly patients with Parkinson’s disease: A study protocol for a randomized controlled trial (AdhCare)**
Trial registration {2a and 2b}.	Trial registration: DRKS00023655 German Clinical Trials Register (DRKS) Date of registration in DRKS: 24 Feb 2021 Last update in DRKS: 22 Mar 2023
Protocol version {3}	Version 2.0, 14 Aug 2023
Funding {4}	This work is funded by the Deutsche Forschungsgemeinschaft (DFG, German Research Foundation) Clinician Scientist Program OrganAge funding number 413668513 by the Interdisciplinary Center of Clinical Research of the Medical Faculty Jena and by a Bundesministerium für Bildung und Forschung grant to HM Mühlhammer, née Zipprich, and T Prell (01GY1804).
Author details {5a}	Sarah Mendorf^1*^, Ulrike Teschner^1^, Thomas Lehmann^3^, Tino Prell^2^, Hannah Mühlhammer^1,2^ 1 Department of Neurology, Jena University Hospital, Jena, Germany 2 Department of Geriatrics, Halle University Hospital, Halle, Germany 3 Institute of Medical Statistics, Computer and Data Sciences, Jena University Hospital, Jena, Germany ^*^Correspondence: sarah.mendorf@med.uni-jena.de; Tel.: +49-3641-9323511
Name and contact information for the trial sponsor {5b}	n/a, as the study was not sponsored
Role of sponsor {5c}	n/a, as the study was not sponsored. The funders had no role in the study design, decision to publish, or writing of the manuscript

## Introduction

### Background and rationale {6a}

Poor adherence is a major issue in health care and is associated with increased morbidity, mortality, and immense costs for the health care system [1–4]. The treatment of chronic disorders usually includes the long-term use of pharmacotherapy and nonpharmacological therapy. However, their complete benefits are often not achieved because approximately 50% of patients either do not take medications as prescribed or do not follow recommendations (termed nonadherence) [5].

Because of demographic changes, the burden of neurological diseases in elderly patients in Germany is increasing, with a critical exacerbation of the problem of nonadherence, as the World Health Organization (WHO) quoted, “increasing the effectiveness of adherence interventions may have a far greater impact on the health of the population than any improvement in specific medical treatments” [6].

In the geriatric population, nonadherence contributes to adverse drug events, increased length of hospital stay and readmissions to hospitals, and lower quality of life [1, 2]. However, physicians often do not routinely enquire about patients’ adherence and are therefore unaware of the extent of patients’ nonadherence to medication.

Many factors contribute to nonadherence [7]. Nonadherence is a dynamic process and may be intentional (when the patient deliberately decides not to follow the recommended treatment) or unintentional (when the patient cannot follow the recommendation). Furthermore, nonadherence often occurs after discharge from the hospital [8–10]. Because the reasons for nonadherence are complex and diverse, interventions to improve adherence must be multifactorial and in the best case trans-sectoral. Although many interventions for increasing adherence have been tested, the best methods to improve adherence remain unclear [11]. Moreover, the available data are mostly restricted to internal medicine. However, it is reasonable to assume that effective intervention strategies must consider the distinct reasons for nonadherence (e.g., a patient who intentionally modifies their medication will not benefit from pill reminders) [12, 13].

More than 20% of adults aged ≥ 60 years experience a mental or neurological disorder (excluding headache disorders), and 6.6% of all disabilities (disability-adjusted life years) among adults aged ≥ 60 years are attributed to neurological and mental disorders [14]. Therefore, evidence-based data are urgently needed to improve adherence in neurogeriatric patients.

Based on the existing data from our large observational trial in the Department of Neurology of the University Hospital Jena, this project will develop a complex intervention to improve adherence in elderly patients with PD. The novel aspect of this intervention is to apply tailored interventions depending on the main personal reason for nonadherence. These personal reasons will be assessed using a detailed adherence self-report, named German Stendal Adherence with Medication Score (SAMS), and include modifications of medication without consulting the physician, forgetting to take the medication, and lack of knowledge about the prescribed medication [15–18]. The following questions will be addressed by the project:

- Can a specific intervention with either behavioral or educational strategy improve motor function and adherence?
- How are these interventions transduced and recognized by the various players in the health care system?

It has been shown that the choice of intervention depends on the feasibility and availability [13]. In this study, one of the key guiding principles is that the intervention is clinically practical and therefore implementable and can later easily be transferred into practice.

#### Objectives {7}

The objective of this study is to improve or maintain motor function by using a tailored intervention to improve adherence to medication in elderly patients with PD.

#### Trial design {8}

This study is based on the NeuGerAd study, a longitudinal observational study of adherence in older adults with neurological disorders [19]. The AdhCare trial is designed as a randomized, controlled, patient, observer, and data analyst-blinded superiority trial with two parallel groups and a primary endpoint of motoric function using UPDRS II and III. It is conducted to test the efficacy of AdhCare, a tailored intervention to enhance adherence compared with that achieved using routine care. The participants are assessed at baseline and at 3 and 6 months of follow-up.

A total of 130 participants are randomized in a 1:1 ratio: intervention in the AdhCare arm (*n* = 64) or control group with standard of care (*n* = 64).

## Methods: participants, interventions, and outcomes

### Study setting {9}

The study is conducted in the Department of Neurology at the University Hospital Jena, Germany.

Patients with PD presenting at our outpatient or inpatient movement disorder unit are included. All patients aged ≥ 60 years with PD are screened for eligibility and recruited after obtaining informed consent. Following our experience with similar studies in this cohort, the willingness of patients to participate in the study is high because there are no harmful interventions planned.

### Eligibility criteria {10}

The inclusion criteria are as follows: elderly patients (age > 60 years) with PD according to the Movement Disorder Society (MDS) criteria, self-management of medication, Montreal Cognitive Assessment (MoCA) ≥ 19, and nonadherence according to SAMS (SAMS ≥ 1).

The SAMS score serves as a basis for comparison and is an established questionnaire utilized to assess nonadherence. A threshold of ≥ 1 point is chosen to determine nonadherence. A score of 1 or higher is indicative of nonadherence, with the severity increasing as the score increases, leading to greater clinical significance.

The exclusion criteria are as follows: MoCA < 19, acute psychotic symptoms, delirium, no active involvement in drug management (e.g., bedridden in a nursing home), and inability to provide informed consent.

### Who will take informed consent? {26a}

The participants are screened by trained personnel and informed about the study by the subinvestigator if meeting the inclusion criteria. Informed consent is given in writing.

### Additional consent provisions for collection and use of participant data and biological specimens {26b}

N/a, as no biological specimens or data relating to this are collected.

### Interventions

#### Explanation for the choice of comparators {6b}

We chose a comparison and control arm to review the effect of the tailored intervention. Eligible Patients are randomized and either included in the control arm called AdhCare, where participants receive an additional tailored intervention according to their nonadherence pattern, or include in the comparison arm, where they do not receive any additional tailored intervention apart from the regular education sessions (standard of care).

#### Intervention description {11a}

Participants with any degree of nonadherence according to SAMS (SAMS ≥ 1) are randomized to the control arm or the AdhCare intervention arm. As it has been shown that implementation of fewer intervention strategies (in one patient) leads to better adherence outcomes [11], our approach is a tailored intervention that is adapted to the main reason for the individual patients’ nonadherence according to a self-reported adherence measure. Therefore, depending on the main reason for nonadherence according to SAMS (modifications, missing knowledge, and forgetfulness), the participants in the AdhCare arm receive a cognitive- or behavioral-focused intervention (Table 1).

**Table 1:** Tailored intervention

Reason for nonadherence	Approach/focus	Particulars
1 Modifications	Educational/alternating strategies, e.g., adjusting the medication regimen	Addressing barriers Physician providing further treatment strongly involved
2 Missing knowledge	Informational/educational strategies to enhance knowledge, also about disease-related risks	Further sources of information are named
3 Forgetfulness	Behavioral: habit-based medication-taking reminders	Strong involvement of a life partner, if possible

The following interventions are provided to the AdhCare (intervention) group:

- A standardized, informational talk to improve patient empowerment to take responsibility and control of their own health. Depending on the main reason for their nonadherence, we, among other things, perform the following interventions:
  1. Modifications: explain the medication, discuss barriers, and strive for patient empowerment to discover ways to improve the medication regimen and emphasize different ways of dealing with emerging problems other than cutting out on medications for instance
  2. Missing knowledge: explain the medication and its indications, provide information about individual risks, and indicate other sources of information
  3. Forgetfulness: present and apply habit-based techniques to help the patient remember to take the medication and strive for patient empowerment to improve the medication regimen
- A written summary of the most significant aspects for their individual improvement of adherence.
- Their physician, who continue to provide treatment, are contacted and informed about the study, individual patients’ adherence problems and barriers, and possible strategies to minimize nonadherence in the particular patient.
- A follow-up telephone call to reinforce the respective focus about the medications and adherence after 2 weeks.

The control group received standard information about the course and therapy of PD.

#### Criteria for discontinuing or modifying allocated interventions {11b}

Whenever a participant expresses the desire to terminate the intervention or withdraws their consent, a discontinuation or modification of the assigned interventions is facilitated.

#### Strategies to improve adherence to interventions {11c}

Strategies to improve adherence to intervention protocols are not required because improving the adherence is the objective of the study and is measured in the participants. Patients are being called in advance by trained personnel to remind them of their appointments, so that the intervention and follow-ups could be carried out. Also, the collection of multiple questionnaires is facilitated by contacting patients by telephone and guiding them through the assessment.

#### Relevant concomitant care permitted or prohibited during the trial {11d}

There is no concomitant care/treatment allowed or prohibited during the study. We conscientiously inquire about and document the treatments that patients receive in order to be able to make statements regarding adherence (improvement) in this regard as well.

#### Provisions for post-trial care {30}

Arrangements for post-study care and compensation for individuals harmed by participation in the study are not necessary because of a cognitive- or behavioral-focused intervention.

### Outcomes {12}

#### Primary outcome

Because the overall goal is to improve patient-reported outcomes, the primary endpoint is motor function, which is assessed using the combined MDS revised Unified Parkinson’s Disease Rating Scale (MDS-UPDRS) II and MDS-UPDRS III after 6 months in the control versus AdhCare arm (intent-to-treat).

#### Secondary outcome

The secondary outcomes are as follows: self-reported adherence (SAMS), medication adherence scores, and number of primary care visits (Table 2). Moreover, we qualitatively evaluate the perception of intervention and barriers and facilitators for the implementation.

**Table 2:** Study measures

	**Tool**	**Baseline**	**3 months**	**6 months**
Descriptive data: sociodemographic	Sex, age, education, income, employment, health insurance, housing	x		
Descriptive data: medical	Reason for admission to the hospital, inpatient or outpatient treatment, ICD-10 diagnoses, prescribed medication list, over-the-counter medication, medication actually taken, health service utilization			
Descriptive data: Parkinson motor	Hoehn and Yahr stage, MDS-UPDRS II, III, IV	x	x	x
Descriptive data: Parkinson nonmotor	MDS-UPDRS I, MoCA, PHQ-9, PDQ-8, GAI	x		x
Descriptive data: adherence	SAMS, change of medication within the 6-month period, difficulties in managing, handling, and swallowing pills	x		x
Descriptive data: comprehensive geriatric assessment	In addition to the aforementioned assessment tools, CIRS, IADL, visual acuity, hearing acuity, urinary incontinence, psychosocial circumstances, body mass index, hospital stay within the 6-month period	x		
Primary outcome: motor function	MDS-UPDRS II, III		x	x
Secondary outcome: self-reported adherence	SAMS			x
Secondary outcome: medication adherence scores	The proportion of self-reported number of pills taken among the number of pills prescribed by the doctor The proportion of the counted number of pills taken among the number of pills prescribed by the doctor		x	x
Secondary outcome: health care access	The number of primary care visits		x	x
Secondary outcome: adherence via medication subscription	The number and frequency of medication prescribed in the 6-month period and prescriptions redeemed at the pharmacy			x
Secondary outcome: qualitative	Perception of intervention and barriers and facilitators for the implementation			x

*ICD* International Statistical Classification of Diseases and Related Health Problems, *MDS-UPDRS* Movement Disorder Society revised Unified Parkinson’s Disease Rating Scale, *MoCA* Montreal Cognitive Assessment, *PHQ-9* Patient Health Questionnaire, *PDQ-8* Parkinson’s Disease Questionnaire, *GAI* Geriatric Anxiety Inventory, *SAMS* Stendal Adherence with Medication Score, *CIRS* Cumulative Illness Rating Scale, *IADL* Instrumental activities of daily living

#### Participant timeline {13}

At baseline and follow-up, the participants complete a staff-administered assessment and a self-report paper-based assessment. These assessments are summarized in Table 2. The time schedule is summarized in Table 3.

**Table 3:** Time schedule

Time point	Study period			
	Enrollment, *-t*_*1*_	Allocation, 0	Post-allocation, *3 months*	Close-out, *6 months*
Enrollment				
Eligibility screen	X			
Informed consent	X			
Allocation		X		
Interventions				
*[AdhCare]*		X Cognitive- or behavioral-focused intervention		
*[Control]*		Standard information about the course and therapy		

#### Sample size {14}

We take as clinically relevant a difference of 5 points on the MDS-UPDRS II-III scale [20]. The group sample (control and comparison arm) sizes of 51 and 51 achieve 80.750% power to reject the null hypothesis of equal means when the population mean difference is 5.0 with a standard deviation for both groups of 8.8 and with a significance level (alpha) of 0.050 using a two-sided two-sample equal-variance *t*-test. The assumed values are based on the literature (see above [20]) and clinical experience. Considering 25% dropout, the final sample size is 64 participants per arm.

#### Recruitment {15}

To maximize participant enrollment, several strategies are being employed, including screening of both inpatients and outpatients of our movement disorder unit in the Department of Neurology at the University Hospital Jena, Germany, to capture a wider range of participants with varying medical conditions, severity levels, and treatment settings. Also, the first patient information is taking place through the attending physicians of our movement disorder unit. During this first contact, the advantages that the study brings to the participants are highlighted.

### Assignment of interventions: allocation

#### Sequence generation {16a}

A block randomization with the help of computer-generated random numbers is performed to ensure that the number of participants in the study groups is approximately equal. Outpatients and inpatients are randomized separately.

#### Concealment mechanism {16b}

Allocation concealment is ensured as the computer-generated randomization code will not be released until the patient has been recruited into the trial. This serves to avoid a selection bias [21].

#### Implementation {16c}

The subinvestigators perform computerized randomization once the patient has consented to participate and then assign the patient to the treatment group accordingly.

### Assignment of interventions: blinding

#### Who will be blinded {17a}

A triple-blinded design is used. The participants will not be aware if they are in the control or AdhCare group. The study personnel assessing the outcome parameter as well as the data analyst are blinded as well.

#### Procedure for unblinding if needed {17b}

The persons administering the cognitive or behavioral intervention are not blinded, so there will be no unblinding. In later phases, as well as at the scheduled follow-up, unblinding is not required because all patients, regardless of group, receive support and assistance for adverse events and problems that arise (which may or may not be due to the intervention).

### Data collection and management

#### Plans for assessment and collection of outcomes {18a}

In the study, paper questionnaires are administered. Trained staff collects PD-related data and data on remaining pill counts and verifies the test results. Table 4 provides an overview of the questionnaires used.

**Table 4:** Overview of the used questionnaires

Score	Cronbach’s alpha	Validity (correlation coefficient *r*)
GAI [22]	0.91	0.57–0.70^a^
MDS-UPDRS [23]	0.79–0.93 across parts	0.76–0.96 across parts^a^
MoCA [24]	0.83	0.61^a^
PDQ-8 [25]	0.80	0.47–0.61^b^
PHQ-9 [26]	0.89	0.55^b^

^a^Concurrent validity

^b^Construct validity

#### Plans to promote participant retention and complete follow-up {18b}

Plans to promote participant retention and full follow-up include detailed education of participants about the study timeline and number of consults. They get written information about their next scheduled appointments and assignments. In addition, our study team is instructed to promote successful study completion through their communication style during data collection. Also, the study team contacts participants to ensure data collection via telephone.

#### Data management {19}

A comprehensive data management plan is implemented to ensure data quality, security, and storage. This includes using standardized templates and guidelines for data entry, conducting regular quality checks and validation procedures, and implementing strict protocols for data security and storage. Data are collected by trained study staff and entered into a dedicated study database (by the same person). This guarantees a secure and direct transfer of data to the study office without any intermediary steps. Additionally, regularly conducted checks ensure the detection of any potential duplicate records and verification of value ranges.

#### Confidentiality {27}

Personal information about potential and enrolled participants is collected, shared, and retained by means of an identifier so that patients can only be matched to the identifier by the study investigators during the study. After completion of the study, only the data with the identifier are used to maintain confidentiality. The data are stored in a password-protected manner on the clinic’s internal drive. The individual trial identification numbers are numbered consecutively in ascending order starting with AC001.

Ensuring the appropriate level of anonymity, confidentiality, and de-identification when maintaining human subject data is crucial for reducing the risk potential for participants, researchers, and the university in alignment with the General Data Protection Regulation (GDPR) of the European Union. All trial data are recorded, processed, handled, and stored without disclosing the personal data of the subjects, so that they can be accurately reported, interpreted, and verified, while protecting the confidentiality of the records and the personal data of the subjects in accordance with the applicable law on the protection of personal data. Appropriate technical and organizational measures are in place to safeguard processed information and personal data from unauthorized or unlawful access, disclosure, dissemination, alteration, or destruction, including accidental loss, particularly when the processing entails transmission over a network.

#### Plans for collection, laboratory evaluation, and storage of biological specimens for genetic or molecular analysis in this trial/future use {33}

Plans for collection, laboratory evaluation, and storage of biological samples for genetic or molecular analyses are not necessary for the study because no biological samples are collected.

## Statistical methods

### Statistical methods for primary and secondary outcomes {20a}

Analysis will take place after full recruitment and follow-up. There are no planned interim analyses for efficacy. Statistical analysis will be tested at the 2-sided 5% significance level with any estimates displayed with 95% confidence intervals (CIs). The mean, standard deviation, and any other statistics other than quantiles will be reported to one decimal place greater than the original data. Quantiles, such as median, or minimum and maximum will use the same number of decimal places as the original data. All outcomes will be presented using descriptive statistics: normally distributed data by the mean and standard deviation (SD) and skewed distributions by the median and interquartile range (IQR). Binary and categorical variables will be presented using counts and percentages.

Baseline characteristics of the study population will be summarized separately within each randomized group using means (with standard deviations), medians (with interquartile ranges), and numbers (with percentages) where appropriate. Baseline characteristics will also be presented for dropouts and completers within each treatment group. Similarly, the primary and secondary outcomes at baseline and all follow-up by treatment groups will be described.

Further analyses will be conducted on all the primary and secondary outcomes. These will be performed based on the intention-to-treat principle and will utilize all available follow-up data from all randomized participants. For the main analysis of treatment effects, crude change in outcomes for both groups from baseline to follow-up will be provided as the mean change scores with standard deviations or proportions, and the corresponding 95% confidence interval (CI) will be presented. The primary outcome (motor function) will be analyzed with a linear mixed effects model. Study subjects will be considered as random effects. Treatment (control or AdhCare group), month (baseline and 3 and 6 months), setting (outpatient and inpatient), and the interaction of treatment with months will be considered as fixed effects. The baseline value of the motor function will be included as a covariate.

Secondary continuous outcomes will be analyzed analogously. However, there will be no adjustment for multiplicity of testing among effect estimates and corresponding 95% CIs for nonprimary outcomes, which therefore should be interpreted cautiously as hypothesis-generating results.

### Interim analyses {21b}

Interim analyses are not included in this study. Early discontinuation is also not planned due to the study design.

### Methods for additional analyses (e.g., subgroup analyses) {20b}

Additional analyses are not planned.

### Methods in analysis to handle protocol nonadherence and any statistical methods to handle missing data {20c}

The data are captured in the electronic case report form and will be transferred anonymously from this to the statistician for processing (i.e., statistical analysis). Automated checks will be implemented, and manual checks will be done against the data to ensure completeness and consistency of data. The database and check programming will be validated prior to implementation. Data identified as erroneous or inconsistent, or key data that are missing, will be referred to the subinvestigators and to the principal investigator for clarification. After these forms are returned, the data will be corrected as required. All modifications to the data will be logged in the audit trail. Prior to the closure of the database, an audit of the raw data will be performed. All errors detected during the audit will be corrected prior to database closure. Visual and computerized methods of data validation will be applied in order to ensure accurate, consistent, and reliable data for the subsequent statistical analysis. These procedures aim to detect out-of-range values, contradictory data, abnormal evolutions over time, and possible protocol deviations (eligibility criteria, time, and medication adherence, etc.).

The sensitivities of all treatment effect estimates to missing outcome data will be explored; these models will explore the robustness of the treatment estimates to whatever small amount of missing data there is. The main analysis will use all available data that we believe are valid under the assumption of missing at random (see primary outcome analysis above). We will then use a suite of sensitivity analysis to explore the robustness of the primary analysis to departures from assumptions, including all randomized participants. If required, sensitivity analyses will include multiple imputation and imputing a range of values for missing data under missing not at random assumptions.

### Plans to give access to the full protocol, participant-level data, and statistical code {31c}

Granting public access to the full protocol, participant-level data set, and statistical code is not currently anticipated but can all be obtained from the author.

### Oversight and monitoring

#### Composition of the coordinating center and trial steering committee {5d}

The research team encompasses a range of expertise, including behavioral and medical management, research design, multicenter data collection, epidemiology and biostatistics, patient engagement, practice transformation, continuing education, quantitative outcomes assessment, and qualitative analysis of processes and outcomes. We did not establish a trail steering committee or Stakeholder and Public Involvement Group in the context of this study.

#### Composition of the data monitoring committee, its role, and reporting structure {21a}

A data monitoring committee is not necessary, and not required by the local ethics committee, due to the short duration and the known minimal risks in this kind of cognitive- and behavioral-focused intervention.

#### Adverse event reporting and harms {22}

Inadequate knowledge, skills, and experience among trained study staff can lead to adverse events such as personal insult, frustration, loss of motivation, emotional distress, and loss of trust being linked to inappropriate practices. If participants report adverse events of any kind, it will be recorded, assessed internally by the team to prevent future adverse events, and reported as part of the planned publication.

#### Frequency and plans for auditing trial conduct {23}

Because there are no sponsors for the study, information on the frequency and procedures for possible review of the study process and the independence of this process from the investigators and sponsors is not required.

However, the project management group meets once a week to discuss the current state of data collection and address potential problems and difficulties. In addition, the trial steering group with experts of different disciplines will meet regularly, initially every month and during the course of the study quarterly, or semi-annually unless unscheduled necessary.

#### Plans for communicating important protocol amendments to relevant parties (e.g., trial participants, ethical committees) {25}

The study is conducted by the staff in accordance with the submitted study protocol. Changes to the study protocol regarding study objectives, study design, eligibility criteria, sample size, or significant changes affecting study conduct, potential benefit, or participant safety will be approved in advance by the study management. Subsequently, the proposed changes will be submitted to the local ethics committee of Jena University Hospital and the relevant regulatory authority for approval before they are implemented. In cases where there is an immediate risk to study participants or unavoidable medical reasons, a change will be implemented prior to regulatory approval. Participants will be notified of any changes to the study and will sign an updated informed consent form reflecting these changes. All deviations from the protocol will be carefully documented in the original records and appropriate reporting of noncompliance will be made.

#### Dissemination plans {31a}

At the end of the study, a final report will be prepared. In addition, we will actively disseminate our results through publications in high-ranking scientific peer-reviewed international and national journals. All results will be reported with reference to the study protocol. Target journals that might be of interest are open-access journals like Frontiers, BioMed Central, or *Journal of Clinical Medicine*. Further dissemination will be achieved by the project itself (via practitioners), by including professional societies (educational events) and by providing informative literature and handouts/flyers to physicians. A meeting with the practicing neurologists and general practitioners is planned. The goal is to translate and transfer our results into health care and to implement recommendations to improve communication between health care providers after the project is completed. In principle, knowledge will be shared by all partners. Participants are asked at the beginning of the study if they want to be informed about the results. If this is the case, information will be provided by telephone or email. In addition, the results will be published after completion of the follow-up.

With regard to public dissemination, we will use the Jena University Hospital website to set up a German language webpage to provide information on the study and to report on the study progress and outcomes. The website will give access to all study materials including the intervention program after the trial. The homepage will address patients, caregivers, researchers, clinicians, and health care providers. Furthermore, we will actively disseminate our results through press reports released via the Jena University Hospital Press and Public Relations Office. We will also present the results to relevant stakeholders such as medical associations and professional societies at meetings, symposia, and poster presentations. In particular, we will organize events for patients and caregivers in order to sensitize them to this topic and to provide ways to reduce barriers of implementation. We will organize regular meetings with the self-help groups and patients with distinct neurological disorders (so-called Patienten-Akademie) in order to inform them about the current developments in the study. We will organize a closing event for the involved physicians to present and discuss the project results.

## Discussion

This protocol presents the design of a randomized controlled trial to evaluate the efficacy of a tailored intervention to improve medication adherence in elderly patients with PD. The AdhCare study has several relevant features. First, we focus on the elderly population. This is because the prevalence of chronic disorders and polypharmacy is high in this population, with a high risk for nonadherence. In addition to testing the efficacy of the intervention, we provide information about the mechanisms and reasons for nonadherence. The results of this study will provide valuable information for health professionals and policymakers on the effectiveness of interventions in elderly patients with PD. The key features of the AdhCare trial are as follows: (i) a carefully characterized target group; (ii) a tailored intervention based on theory and evidence from earlier studies; (iii) a quality-assured delivery enabled by training, ongoing supervision, and protocols; and (iv) objective geriatric assessments and measurements of functional parameters as well as a self-report of functional status, mood, and quality of life.

## Trial status

Protocol version: version 2.0, 14 August 2023.

Start of recruitment: 26 April 2021.

End of recruitment: 03 March 2023.

Planned study completion date: 30 September 2023.

Due to the COVID-19 pandemic, we had to allocate our available personnel extensively to the execution of the study. This resulted in staffing shortages and increased workload, which caused a delay in processing papers and submitting this protocol. Furthermore, staff shortages due to illness, the need to assist in other areas of the health care facility, and maternity leave are reasons that also contributed to the delay.

## Data Availability

The authors have access to the final data set of the study. Any data needed to support the protocol can be provided upon request. Participant information materials and consent forms are available from the corresponding author upon request. The data sets and statistical code analyzed in the current study are available from the corresponding author upon request, as is the full protocol.

## Abbreviations

CIRS: Cumulative Illness Rating Scale
GAI: Geriatric Anxiety Inventory
IADL: Instrumental activities of daily living
MDS: Movement Disorder Society
MDS-UPDRS: Movement Disorder Society revised Unified Parkinson’s Disease Rating Scale
MoCA: Montreal Cognitive Assessment
PHQ-9: Patient Health Questionnaire
PD: Parkinson’s disease
PDQ-8: Parkinson’s Disease Questionnaire
SAMS: Stendal Adherence with Medication Score
WHO: World Health Organization
